# Alloying Platinum with Gold

**Published:** 1906-07

**Authors:** J. Herbert Barton

**Affiliations:** St. John, N. B.


### ALLOYING PLATINUM WITH GOLD.

By Mr. J. Herbert Barton, St. John, N. B.

Pure platinum cannot be melted by the ordinary gas blowpipe, nor can it be directly alloyed with gold, excepting at a very high temperature maintained for a considerable length of time; but there are some metals with which it can be alloyed, and once alloyed with these it readily fuses with gold.

In some experiments conducted by myself, I attempted to fuse with the ordinary gas blowpipe equal parts of platinum and gold; platinum and silver, and platinum and copper.

In the case of the platinum and gold, the platinum could not be induced to melt at all; the gold merely melted and flowed over and around it.

In the case of the platinum and silver, the platinum partly fused and made an indifferent alloy, some pieces only partly melting.

But with the copper combination, the platinum fused thoroughly, making a homogenous piece of metal throughout. Once alloyed with copper it readily fused and mixed with gold.

Applying this to the making of platinized gold, I take my alloy, consisting of platinum, silver and copper, and thoroughly melt, then add my gold. I have found the following to be a splendid formula:

| | |
|---|---|
| Pure gold | 18 parts. |
| Pure platinum | 2 parts. |
| Pure silver | 2 parts. |
| Pure copper | 2 parts. |

Place the platinum, silver, and copper on a hollowed piece of charcoal on the melting-frame, and using borax for a flux, direct a good hot blast of flame from any good blowpipe, with foot bellows (I use a Melotte's pipe with No. 7 Buffalo foot bellows), and the metals will soon fuse; get the heat as intense as possible, and hold it there for a few minutes, then add your gold and remelt, shaking or mixing with a piece of wood to insure a good mix. Pour into an ingot, or it may be left to cool on the coal, after which it can be rolled into plate or drawn into wire.

This metal is second only to steel in hardness and elasticity, and has a high fusing-point. It is much superior to the clasp metal sold at some of the depots, and is excellent for making clasps for partial rubber dentures, bands, ribbons, wires, screws, etc., for regulating applicances, posts for Richmond crowns, etc. —*Cosmos.*

